# Supplementary material for: HIVprotI: an integrated web based platform for prediction and design of HIV proteins inhibitors
Source: J Cheminform. 2018 Mar 9;10:12. doi: 10.1186/s13321-018-0266-y (PMC5845081; doi:10.1186/s13321-018-0266-y)
Supplement: Supplementary file 2 — Additional file 2. Source code of HIVProtI web server. [file 13321_2018_266_MOESM2_ESM.zip › HIVprotI_Source-code/hivprotI/weka-3-6-6/doc/weka/filters/Filter.html]

Filter


---


|  |  |  |
| --- | --- | --- |
| **Overview** \| **Package** \| **Class** \| **Tree** \| **Deprecated** \| **Index** \| **Help** \| **Weka's home** | |  |
| **PREV CLASS**   **NEXT CLASS** | **FRAMES**    **NO FRAMES**     **All Classes** |  |
| SUMMARY: NESTED \| FIELD \| CONSTR \| METHOD | DETAIL: FIELD \| CONSTR \| METHOD |  |


---


## weka.filters Class Filter

```
java.lang.Object
  weka.filters.Filter
```

**All Implemented Interfaces:**: java.io.Serializable, CapabilitiesHandler, RevisionHandler

**Direct Known Subclasses:**: AbstractTimeSeries, Add, AddCluster, AddExpression, AddID, AddNoise, AddValues, AllFilter, AttributeSelection, ChangeDateFormat, ClassOrder, ClusterMembership, Copy, Discretize, FirstOrder, MakeIndicator, MergeTwoValues, MultiInstanceToPropositional, NominalToBinary, NominalToBinary, NominalToString, NonSparseToSparse, Normalize, NumericTransform, Obfuscate, PotentialClassIgnorer, PrincipalComponents, PropositionalToMultiInstance, Randomize, RandomProjection, Remove, RemoveFolds, RemoveFrequentValues, RemoveMisclassified, RemovePercentage, RemoveRange, RemoveType, RemoveUseless, RemoveWithValues, Reorder, Resample, Resample, ReservoirSample, SimpleFilter, SMOTE, SparseToNonSparse, SpreadSubsample, StratifiedRemoveFolds, StringToNominal, StringToWordVector, SwapValues

---

``` public abstract class Filter extends java.lang.Object implements java.io.Serializable, CapabilitiesHandler, RevisionHandler ```

An abstract class for instance filters: objects that take instances
as input, carry out some transformation on the instance and then
output the instance. The method implementations in this class
assume that most of the work will be done in the methods overridden
by subclasses.

A simple example of filter use. This example doesn't remove
instances from the output queue until all instances have been
input, so has higher memory consumption than an approach that
uses output instances as they are made available:

```` ```
  Filter filter = ..some type of filter..
  Instances instances = ..some instances..
  for (int i = 0; i < data.numInstances(); i++) {
    filter.input(data.instance(i));
  }
  filter.batchFinished();
  Instances newData = filter.outputFormat();
  Instance processed;
  while ((processed = filter.output()) != null) {
    newData.add(processed);
  }
  ..do something with newData..
``` ```` 

**Version:**
:   $Revision: 6833 $

**Author:**
:   Len Trigg (trigg@cs.waikato.ac.nz)

**See Also:**: Serialized Form

---

**Constructor Summary**

`Filter()`


| **Method Summary** | |
| --- | --- |
| `static void` | `batchFilterFile(Filter filter, java.lang.String[] options)`             Method for testing filters ability to process multiple batches. |
| `boolean` | `batchFinished()`             Signify that this batch of input to the filter is finished. |
| `static void` | `filterFile(Filter filter, java.lang.String[] options)`             Method for testing filters. |
| `Capabilities` | `getCapabilities()`             Returns the Capabilities of this filter. |
| `Capabilities` | `getCapabilities(Instances data)`             Returns the Capabilities of this filter, customized based on the data. |
| `Instances` | `getOutputFormat()`             Gets the format of the output instances. |
| `java.lang.String` | `getRevision()`             Returns the revision string. |
| `boolean` | `input(Instance instance)`             Input an instance for filtering. |
| `boolean` | `isFirstBatchDone()`             Returns true if the first batch of instances got processed. |
| `boolean` | `isNewBatch()`             Returns true if the a new batch was started, either a new instance of the filter was created or the batchFinished() method got called. |
| `boolean` | `isOutputFormatDefined()`             Returns whether the output format is ready to be collected |
| `static void` | `main(java.lang.String[] args)`             Main method for testing this class. |
| `static Filter[]` | `makeCopies(Filter model, int num)`             Creates a given number of deep copies of the given filter using serialization. |
| `static Filter` | `makeCopy(Filter model)`             Creates a deep copy of the given filter using serialization. |
| `int` | `numPendingOutput()`             Returns the number of instances pending output |
| `Instance` | `output()`             Output an instance after filtering and remove from the output queue. |
| `Instance` | `outputPeek()`             Output an instance after filtering but do not remove from the output queue. |
| `boolean` | `setInputFormat(Instances instanceInfo)`             Sets the format of the input instances. |
| `java.lang.String` | `toString()`             Returns a description of the filter, by default only the classname. |
| `static Instances` | `useFilter(Instances data, Filter filter)`             Filters an entire set of instances through a filter and returns the new set. |
| `static java.lang.String` | `wekaStaticWrapper(Sourcable filter, java.lang.String className, Instances input, Instances output)`             generates source code from the filter |

**Methods inherited from class java.lang.Object**

`equals, getClass, hashCode, notify, notifyAll, wait, wait, wait`

**Constructor Detail**

### Filter

```
public Filter()
```


**Method Detail**

### isNewBatch

```
public boolean isNewBatch()
```

:   Returns true if the a new batch was started, either a new instance of the
    filter was created or the batchFinished() method got called.

    :   **Returns:**: true if a new batch has been initiated **See Also:**: `m_NewBatch`, `batchFinished()`

---


### isFirstBatchDone

```
public boolean isFirstBatchDone()
```

:   Returns true if the first batch of instances got processed. Necessary for
    supervised filters, which "learn" from the first batch and then shouldn't
    get updated with subsequent calls of batchFinished().

    :   **Returns:**: true if the first batch has been processed **See Also:**: `m_FirstBatchDone`, `batchFinished()`

---


### getCapabilities

```
public Capabilities getCapabilities()
```

:   Returns the Capabilities of this filter. Derived filters have to
    override this method to enable capabilities.

    :   **Specified by:**: `getCapabilities` in interface `CapabilitiesHandler`
    :   **Returns:**: the capabilities of this object **See Also:**: `Capabilities`

---


### getRevision

```
public java.lang.String getRevision()
```

:   Returns the revision string.

    :   **Specified by:**: `getRevision` in interface `RevisionHandler`
    :   **Returns:**: the revision

---


### getCapabilities

```
public Capabilities getCapabilities(Instances data)
```

:   Returns the Capabilities of this filter, customized based on the data.
    I.e., if removes all class capabilities, in case there's not class
    attribute present or removes the NO\_CLASS capability, in case that
    there's a class present.

    :   **Parameters:**: `data` - the data to use for customization **Returns:**: the capabilities of this object, based on the data **See Also:**: `getCapabilities()`

---


### setInputFormat

```
public boolean setInputFormat(Instances instanceInfo)
                       throws java.lang.Exception
```

:   Sets the format of the input instances. If the filter is able to
    determine the output format before seeing any input instances, it
    does so here. This default implementation clears the output format
    and output queue, and the new batch flag is set. Overriders should
    call `super.setInputFormat(Instances)`

    :   **Parameters:**: `instanceInfo` - an Instances object containing the input instance structure (any instances contained in the object are ignored - only the structure is required). **Returns:**: true if the outputFormat may be collected immediately **Throws:**: `java.lang.Exception` - if the inputFormat can't be set successfully

---


### getOutputFormat

```
public Instances getOutputFormat()
```

:   Gets the format of the output instances. This should only be called
    after input() or batchFinished() has returned true. The relation
    name of the output instances should be changed to reflect the
    action of the filter (eg: add the filter name and options).

    :   **Returns:**: an Instances object containing the output instance structure only. **Throws:**: `java.lang.NullPointerException` - if no input structure has been defined (or the output format hasn't been determined yet)

---


### input

```
public boolean input(Instance instance)
              throws java.lang.Exception
```

:   Input an instance for filtering. Ordinarily the instance is
    processed and made available for output immediately. Some filters
    require all instances be read before producing output, in which
    case output instances should be collected after calling
    batchFinished(). If the input marks the start of a new batch, the
    output queue is cleared. This default implementation assumes all
    instance conversion will occur when batchFinished() is called.

    :   **Parameters:**: `instance` - the input instance **Returns:**: true if the filtered instance may now be collected with output(). **Throws:**: `java.lang.NullPointerException` - if the input format has not been defined.: `java.lang.Exception` - if the input instance was not of the correct format or if there was a problem with the filtering.

---


### batchFinished

```
public boolean batchFinished()
                      throws java.lang.Exception
```

:   Signify that this batch of input to the filter is finished. If
    the filter requires all instances prior to filtering, output()
    may now be called to retrieve the filtered instances. Any
    subsequent instances filtered should be filtered based on setting
    obtained from the first batch (unless the inputFormat has been
    re-assigned or new options have been set). This default
    implementation assumes all instance processing occurs during
    inputFormat() and input().

    :   **Returns:**: true if there are instances pending output **Throws:**: `java.lang.NullPointerException` - if no input structure has been defined,: `java.lang.Exception` - if there was a problem finishing the batch.

---


### output

```
public Instance output()
```

:   Output an instance after filtering and remove from the output queue.

    :   **Returns:**: the instance that has most recently been filtered (or null if the queue is empty). **Throws:**: `java.lang.NullPointerException` - if no output structure has been defined

---


### outputPeek

```
public Instance outputPeek()
```

:   Output an instance after filtering but do not remove from the
    output queue.

    :   **Returns:**: the instance that has most recently been filtered (or null if the queue is empty). **Throws:**: `java.lang.NullPointerException` - if no input structure has been defined

---


### numPendingOutput

```
public int numPendingOutput()
```

:   Returns the number of instances pending output

    :   **Returns:**: the number of instances pending output **Throws:**: `java.lang.NullPointerException` - if no input structure has been defined

---


### isOutputFormatDefined

```
public boolean isOutputFormatDefined()
```

:   Returns whether the output format is ready to be collected

    :   **Returns:**: true if the output format is set

---


### makeCopy

```
public static Filter makeCopy(Filter model)
                       throws java.lang.Exception
```

:   Creates a deep copy of the given filter using serialization.

    :   **Parameters:**: `model` - the filter to copy **Returns:**: a deep copy of the filter **Throws:**: `java.lang.Exception` - if an error occurs

---


### makeCopies

```
public static Filter[] makeCopies(Filter model,
                                  int num)
                           throws java.lang.Exception
```

:   Creates a given number of deep copies of the given filter using
    serialization.

    :   **Parameters:**: `model` - the filter to copy: `num` - the number of filter copies to create. **Returns:**: an array of filters. **Throws:**: `java.lang.Exception` - if an error occurs

---


### useFilter

```
public static Instances useFilter(Instances data,
                                  Filter filter)
                           throws java.lang.Exception
```

:   Filters an entire set of instances through a filter and returns
    the new set.

    :   **Parameters:**: `data` - the data to be filtered: `filter` - the filter to be used **Returns:**: the filtered set of data **Throws:**: `java.lang.Exception` - if the filter can't be used successfully

---


### toString

```
public java.lang.String toString()
```

:   Returns a description of the filter, by default only the classname.

    :   **Overrides:**: `toString` in class `java.lang.Object`
    :   **Returns:**: a string describing the filter

---


### wekaStaticWrapper

```
public static java.lang.String wekaStaticWrapper(Sourcable filter,
                                                 java.lang.String className,
                                                 Instances input,
                                                 Instances output)
                                          throws java.lang.Exception
```

:   generates source code from the filter

    :   **Parameters:**: `filter` - the filter to output as source: `className` - the name of the generated class: `input` - the input data the header is generated for: `output` - the output data the header is generated for **Returns:**: the generated source code **Throws:**: `java.lang.Exception` - if source code cannot be generated

---


### filterFile

```
public static void filterFile(Filter filter,
                              java.lang.String[] options)
                       throws java.lang.Exception
```

:   Method for testing filters.

    :   **Parameters:**: `filter` - the filter to use: `options` - should contain the following arguments:

---


### batchFilterFile

```
public static void batchFilterFile(Filter filter,
                                   java.lang.String[] options)
                            throws java.lang.Exception
```

:   Method for testing filters ability to process multiple batches.

    :   **Parameters:**: `filter` - the filter to use: `options` - should contain the following arguments:

---


### main

```
public static void main(java.lang.String[] args)
```

:   Main method for testing this class.

    :   **Parameters:**: `args` - should contain arguments to the filter: use -h for help


---


|  |  |  |
| --- | --- | --- |
| **Overview** \| **Package** \| **Class** \| **Tree** \| **Deprecated** \| **Index** \| **Help** \| **Weka's home** | |  |
| **PREV CLASS**   **NEXT CLASS** | **FRAMES**    **NO FRAMES**     **All Classes** |  |
| SUMMARY: NESTED \| FIELD \| CONSTR \| METHOD | DETAIL: FIELD \| CONSTR \| METHOD |  |


---
